# Topics of Public Interest

**Published:** 1917-11

**Authors:** 


### TOPICS OF PUBLIC INTEREST

---

**Status of Internes and Medical Students With Regard to Draft.** Hospital internes and medical students down to the second year may enlist in the Enlisted Reserve Corps, thus anticipating the draft. If already drafted and accepted, they may apply for service in the same. They will thus virtually be exempted from draft for the National Army, although this depends upon the action of the Surgeon General and the Adjutant General. The general policy will be to put such men on waiting orders, internes to report every three months, students at the end of each semester, and to call them into active service at the expiration of a year's interne service or at the close of a college year in case the student is not promoted. This mode of exemption will not apply to internes after a year of interne service. It is required that the medical college be "well recognized," that the interneship shall not be in a sanatorium or private hospital operated for profit, nor in one of less than 50 beds, nor will interneships be recognized if established after May 18, 1917 (the date on which the draft law became effective) unless by reason of a proportionate, bona fide increase in bed capacity of a hospital, nor if entered upon later than Aug. 1, after graduation in medicine. It will be noted that these regulations (established by order of the President) provide throughout for good faith on the part of those directly interested and those in charge of medical schools and hospitals and that they are elastic enough to allow for future emergencies; that they do not allow prolongation of either undergraduates or hospital studies beyond the usual term, for the sake of avoiding service but, on the other hand, are an incentive to good scholarship; that they prevent the nominal or actual election of a medical career as a means of avoiding military service, by applying only to students already matriculated and who have completed the first year of study; that, subject to emergent needs, they allow the completion of the whole course of medical study without interference by the draft. A complicated problem seems to have been ideally solved.

---

**Hospital Provisions.** At cantonments in this country, hospitals will be provided to accommodate about 3% of the troops. Each hospital will have 1000 or more beds, will occupy 60 acres including auxiliary buildings and grounds and will cost about $500,000 for the National Army cantonments. The National Guard camps, being temporary, will have hospitals of lighter construction, without heating and plumbing connections and will cost about $400,000 each. 32 hospitals are projected at present. Abroad, it is estimated that hospital accommodations for 20% of troops should be provided.

---

**Promotions in Medical Dept. of the Army.** There is, at present, no authority for the appointment of a general, except the one Surgeon General so that promotions of colonels have not been possible. Several majors have become lt. colonels and nearly the entire list of captains for 1916 have become majors. A few who were first lieutenants in 1916 are already majors, and many are captains. Since writing above, four colonels have been commissioned brigadier-general.

**Volunteer Enlistment in Regular Army.** In the six months, April-Sept., 218,547 enlistments have been received, bringing the army up to about 300,000 after deducting discharges and deaths.

---

**Death of Insane Patient Under Peculiar Circumstances.** About the first of August, Mrs. Kate Onusczak of Lackawanna went, or was sent to Akron, O., to receive treatment for mental disorder from a midwife. The treatment consisted in packing the patient in a ''bath'' of feathers. She took her baby with her. Some time in September, the local authorities arrested her as insane and her husband went to Akron, secured the baby and returned home. On Sept. 24, the Akron authorities, apparently fearing that the patient would become a public charge, ordered the sheriff to send her home. He, accordingly, packed and secured her in an undertaker's basket. She arrived in Buffalo, extremely depressed physically, filthy, and violent. After a few days' more she died, without having shown indications of violence requiring extreme measures of restraint. Necropsy revealed no adequate cause of death which was ascribed to the exhaustion due to the method of restraint while traveling. It does not seem to us that the use of the carrying basket, under proper limitations, to convey a violently insane patient, can be condemned. Used for a patient not violent and presumably sufficiently in the possesion of her mentality to realize its significance, the mere fact of being placed in a receptacle for dead bodies would be enough to disturb the mind to a very serious degree. Many persons, scarcely even deserving to be stigmatized as hysteric or neurotic and not in any sense insane, would be rendered insane by this procedure alone. With the addition of retention devices and the lack of attention to the ordinary necessity of keeping the patient's bedding in a state of cleanliness, the effect on the psychic state of the patient might well be serious, even fatal as in the present case. Three other questions present themselves: Why should a midwife, in any civilized country, be allowed to treat psychoses, or any other serious condition? We pass over the merits of the feather pack as the exact method of therapeutics by extra-professional practitioners usually involves bizarre procedures. Why was the patient's husband allowed to leave Akron without giving proper guarantee of the patient's care and support? Why should the ''shipping'' method be applied to any human being sick or well, for the sake of avoiding improper public burdens of any kind? In this regard, the case is in analogy with the driving out of tramps, the banishment of prostitutes, the sentence of an undesirable to leave town within 24 hours, or any similar makeshift to avoid local responsibility without reference to the disposal of the case in the interests of the individual or of the public. Some reasonable, efficient co-operative plan ought to be readily devised to cover all cases, allotting the responsibility according to established general principles.

---

**The University of Buffalo** opened its sessions for all departments, Sept. 24. This is the 73d session of the Medical Dept. which, for many years, constituted the entire university. Registration in Med. Dept.: Freshmen 83 (20-25% liable to draft), Sophomores 62, Juniors 40, Seniors 28 (several members of upper classes having voluntarily entered the military service).

---

**Saving of Waste in Garbage.** There is a classic story of a garbage collector who estimated the social standing of his clientele by the elegance of their swill. The State Bureau of Municipal Information has found that the garbage of N. Y. City (Manhattan, Bronx and Brooklyn) was 5,953 cubic yards less in June 1917 than in June 1916; of Buffalo 1,069 tons less; of Rochester 693 tons less; of Syracuse 38 tons less, other cities in somewhat the same proportion. The diminution for July was less than for June. This is ascribed to a relaxation in prudence but it occurs to us that it may really represent an economy by heeding the published warnings to preserve food and to use fresh vegetables as much as possible, both of which procedures would inevitably cause an increase in garbage as such food stuffs contain a large amount of waste matter. The garbage, and waste paper and tins of training camps has been collected and sold so as materially to increase the funds available for extra rations.

---

**Prolificity.** Mrs. Jean Baptiste died in Ottawa, Sept. 28, aged 98. She had 2 sons and 10 daughters; 79 grandchildren; 212 great grandchildren; and, at the time of her death, 28 great great grandchildren.

---

**Death From Exhaust Fumes.** George N. Swan of Buffalo was found dead in his garage, Sept. 30, his absence for two hours having caused alarm. The engine of his car was running and the garage was filled with fumes. We have already called attention to several deaths and several narrow escapes. The exhaust fumes include carbon onoxid and probably a mixture of hydrocarbons and partially oxidized products which are more or less anaesthetic, thus producing unconsciousness.

**Conservation of Drugs.** The Committee of Public Safety of Pennsylvania, has issued a letter, especially to druggists but also applying to physicians. Special attention is called to possible loss by deterioration, of biologic products in general, vaccines, Bulgarian bacillus cultures, and of preparations of ergot, digitalis and strophanthus. It is urged, therefore, that care should be taken not to overstock with such materials.

---

**Books for Soldiers.** The Buffalo Public Library or any of its branches will receive books for soldiers. Discards and poorly printed books are not wanted but a book is not necessarily worthless because it is not brand new. Standard fiction is especially desired but works on history, technical books on aviation, wireless, submarines, automobiles, telegraphy, hygiene, etc., and especially French grammars, dictionaries and lesson books and good scientific books generally are needed. Current magazines, the kind that you would read yourself, are also wanted.

---

**Admission of Women to Medical Courses** is announced by New York University and Harvard. So far as we understand the qualifications, however, the former will admit women only to a two-years' medical preparatory course and the latter will limit its admissions to Radcliffe graduates, offering also a two-years' preparatory course.

---

**The Diazo Test** has been abandoned by the N. Y. City Health Dept. Last year's experience gave 250 positive reactions out of 1700. It does not occur in mild cases of typhoid and does occur more or less frequently in cancer, pneumonia, tuberculosis, various acute fevers and after taking naphthalin, chrysarobin and tannin.

---

**Glycerin,** government chemists announce, can be made from sugar at a cost of about 25 cents a pound as against 90 cents from fats.

---

**American Medical Journals.** The 1917 edition of Polk's Directory lists 190 journals for the U. S. and 12 for Canada (not counting State Health Bulletins and a few similar publications). 45 of those in the U. S. have been discontinued and 20 new ones added since 1914. There have also been numerous changes in editorship, title, and addresses of publication, even from one city to another or to quite distant cities. Many of the discontinuances have been due to merger. The medical journals of the U. S. may be divided as follows:

Organization (A. M. A., State and a few county and interstate) 32. Specialties (several conducted by associations) 62. Sectarian (organization and private) 20. General nature and general circulation 16. General nature but local circulation 50.

---

**Statistics of Army Medical Corps,** Oct. 1. Enlisted personnel over 69,000, as against 6,600 before the war. Medical Reserve Corps, nearly 13,000 commissions accepted. Dental Reserve Corps over 2600 commissions accepted. Sanitary Corps 240. Regular Nurse Corps over 300, as against 230 Mch. 1. Reserve Nurse Corps 1600, as against 227 Mch 1. In the same time, the Surgeon-General's office has increased from 10 officers and 140 clerks and messengers to over 100 officers and over 500 clerks and messengers. The really wonderful thing is not the rapid expansion but the fact that the whole system has not broken down under its own sudden increase. On the contrary, the Medical Department of the Army led by several years in the establishment of a reserve corps, prepared for a possible emergency which general opinion regarded as altogether impossible, up to the maximum degree allowed, and further, elaborated a system which has worked under the enormous strain of necessary sudden increase in numbers all along the line, so nearly to perfection that it has anticipated the actual demands placed upon it and holds in reserve forces for practically every need, several months ahead of present demands. Will the medical profession show its appreciation of its own professional conferes by a further, immediate volunteering of services, so that the Medical Department can not only hold its lead but do so without wasting any of its work or mental force through anxiety about the future? This is not merely an appeal to patriotism but to professional esprit de corps. Without in the least condemning the general plan of the selective draft, let the medical profession show that it is unnecessary.

---

**Medical Reserve for N. Y. State and Erie Co.** According to Captain Van Buren, representing the state branch of the Council on Public Safety, there are about 15,600 practicing physicians in the state, of whom 15% are needed for the reserve, 2350 and of whom about 1900, 12% have been recommended for commission. For Erie Co., the corresponding figures are: 868, 130 and 62—7%. Are we slackers at this end of the State? The Council has laid down the following principles for exemption—moral at present, actual in case of draft: single physician in a village, serving an isolated community; hospital staffs not to be depleted below 50% of medical and surgical attendants; important executive positions, as health commissioner, superintendent of hospital, etc.; 3 or more dependents, 75% or more dependent on physician's earnings; and, of course, advanced age and physical infirmities, female sex of physician. It is estimated from careful study of the state military census that Erie Co. has 503 potentially available men for the Medical Reserve so that the quota of 15% of the total, means more than 25% of this number. It is stated that fully 90% of the Reserve officers have been ordered to duty.

---

**The Training School of the Buffalo Homoeopathic Hospital** held its commencement exercises Oct. 5. The following were graduated: Frances H. Hagar, Helen M. Daley, Marjorie Mulcuhy, Florence O. Benson, Hazel A. Rykert, Glena A. McPhee, Teresa M. Kirsch, Irene H. Neelson, Alice Jane Caley, Margaret S. Bell, Martha Ann Calladine, Helen D. Thomas, Irene G. Clark, Josephine D. Wilcox, Olive R. Cole, Kathryn Curran, Ruth S. Costello, Winifred Stickles.

---

**The Food Supply.** It is a very simple sum in arithmetic to show that, at 2000 calories a day, the per capita food for a year must equal 730,000 calories; at 2500, 912,500; and at 3000, 1,095,000. At a liberal average, we may take the convenient number of one million as representing the caloric ration for a year.

Recent official estimates of the crops for the present fall, in the U. S., reduced to pounds per capita on the basis of 100,000,000 population, are as follows: wheat 395, corn 1792, oats 505, barley 96, rice 20, buckwheat 8; total cereals 2316, net pounds after reducing to flour or meal, etc., with minimum waste, 2,000. Potatoes (allowing 20% for peeling) 216 pounds; sweet potatoes 36 pounds. Beans, (5 principal states only) 9.48 pounds.

These represent in calories per capita: cereals 3,175,000; potatoes 71,190; sweet potatoes 18,780; beans 13,900; total 3,288,870.

Here is a food supply for more than three years for our own people; or for the U. S. and all its European allies except Russia (totaling about 247,000,000 people) for nearly 1½ years. This ignores fresh fruits and vegetables, sugar, meats, butter, milk, cheese, eggs, lard, etc.

Foods of this last group, in storage in the large depots of the U. S., Oct. 1, amounted to 613 million pounds of meats, 8 of lard, 105 of butter, 84 of American cheese, 2½ billion eggs. These figures do not include minor depots nor the current market and retail supplies.

Mr. Hoover has announced that definite measures are to be undertaken to control the price of wheat, flour and bread, as well as other staple foods. The surprising statement is made that the cost of converting wheat into flour and marketing the flour is only 40-60 cents a barrel (196 pounds) or about 1/5 up to 1/3 cent a pound. This low cost is due to the value of by-products and probably is not possible on the theoretic maximum conversion of 90% of wheat into flour or even on the basis of using 4 bushels of wheat (240 pounds) to produce a barrel of flour, which corresponds to a waste of nearly 20%. It is also announced that some kind of ''war bread'' will be marketed at retail for 5 cents a loaf (Full pound?). We heartily endorse everything that Mr. Hoover has said in regard to regulating food prices, except his apology which is somewhat in line with that of the man who explained, when seen at church, that his car had broken down. A government should become paternal at just the moment when it ceases to be fraternal, i.e. when practical experience shows that certain big brothers are the only ones who can carry on large industries and that they exploit their little brothers.

---

**Workman's Compensation Law.** The State Industrial Commission will, hereafter, pass upon all claims for medical services to injured employes and direct the method and amounts to be paid. If the workman applies to his employer or accepts the attendance provided by the latter, any claim will be against the latter. If the workman employs a physician directly, the claim will be against the workman himself but subject to the supervision of the Commission. On the one hand, this will possibly reduce the fees which physicians and surgeons consider just; on the other hand, suits for claims will be rendered unnecessary.

---

**Stenographers and Type Writers Needed.** The U. S. Civil Service Commission, John A. McIlhenny, Pres., Washington, D. C., states that there is urgent need of a large force and our readers are asked to send names.

---

**Nikalgen,** a proprietary preparation probably composed largely of quinine and urea hydrochlorate, has been found efficient in France as a local analgesic, when applied on raw surfaces especially burns. Almost complete relief is obtained in 5-10 minutes and persists 3-24 hours, more than 5-7 applications (by spray or saturated dressing) being rarely necessary. It is noteworthy that in this case, as that of ambrine ethical considerations have not prevented the use of practical measures. It may be still necessary to emphasize that the relief of pain by diminishing shock, facilitates healing.

---

**Exemptions Before Physical Examination of Drafted Men** is the order of the Provost Marshal. This is a matter of common sense under the emergent conditions present, yet it would be a great benefit if the 10,000,000 young men drawn could be given a physical examination.

---

**Belgian Birth and Death Rates.** In the Brussels District, the rates for 1913 were 17:1000 births and 13-7:1000 deaths. For the first half of 1915, the respective rates were 14.3 and 14. For the first half of 1917, 8.5 births to 19.3 deaths. These statistics corroborate the contention which we made at the beginning of the war that the distribution of the population by age and sex would not be changed by military casualties to the extent prophesied, because the deaths of the non-combatant population would be very high as a result of privation. The condition in Belgium is, in many respects extreme, approaching that of stone and bronze age warfares. It would not be at all surprising if, after the war, it were found that the greatest losses of population were noted, not in men of military age, but among the aged and infants.

---

**The Wheat and Rye Crop** of Germany is stated to be 7½ million tons against 13 last year. Assuming the population to be 65 million, this comes to about 404,000 calories per capita, or 40% of that needed from all sources. This is not bad in itself, especially when it is implied that the crop of potatoes is good. However, when we reflect that the U. S. crop of wheat alone is 395 pounds, of rye at least 20, and that of both wheat and rye in Germany only 254 pounds per capita, the difference is striking. It is still more so when we consider that, at a pinch, we could live entirely on our corn crop, so far as cereal food is concerned, and still have enough for feed for stock. In these comparisons, we have presumed that ton in Germany means a metric ton of about 2200 pounds.

---

**Preservation of Practices of Medical Volunteers.** Our scepticism as to the practicability of the methods of fraternal care of practices receives support from the fact that a single issue of the Journal of the A. M. A. contains 8 advertisements for locum tenentes for the period of the war, on a basis of sale, small percentage or rental of equipment.

**The Buffalo General Hospital** formally opened its new private room addition, Oct. 23.

**The N. Y. Skin and Cancer Hospital** announces a course of clinics on diseases of the skin, by Dr. L. Duncan Bulkley, Wednesday afternoons at 4:15, beginning Nov. 7, in the Out-Patient Hall, 2d Ave. and 19th St. Physicians admitted on presentation of professional card.

